# CD44v4 Is a Major E-Selectin Ligand that Mediates Breast Cancer Cell Transendothelial Migration

**DOI:** 10.1371/journal.pone.0001826

**Published:** 2008-03-19

**Authors:** Ke Zen, Dan-Qing Liu, Ya-Lan Guo, Chen Wang, Jun Shan, Ming Fang, Chen-Yu Zhang, Yuan Liu

**Affiliations:** 1 State Key Laboratory of Pharmaceutical Biotechnology, School of Life Sciences, Nanjing University, Nanjing, Jiangsu, China; 2 Jiangsu CDC-Nanjing University Joint Institute of Virology, Nanjing, Jiangsu, China; 3 Program of Cell and Molecular Biology, Department of Biology, Georgia State University, Atlanta, Georgia, United States of America; Illinois Institute of Technology, United States of America

## Abstract

**Background:**

Endothelial E-selectin has been shown to play a pivotal role in mediating cell–cell interactions between breast cancer cells and endothelial monolayers during tumor cell metastasis. However, the counterreceptor for E-selectin and its role in mediating breast cancer cell transendothelial migration remain unknown.

**Methodology/Principal Findings:**

By assessing migration of various breast cancer cells across TNF-α pre-activated human umbilical vein endothelial cells (HUVECs), we found that breast cancer cells migrated across HUVEC monolayers differentially and that transmigration was E-selectin dependent. Cell surface labeling with the E-selectin extracellular domain/Fc chimera (exE-selectin/Fc) showed that the transmigration capacity of breast cancer cells was correlated to both the expression level and localization pattern of E-selectin binding protein(s) on the tumor cell surface. The exE-selectin/Fc strongly bound to metastatic MDA-MB-231, MDA-MB-435 and MDA-MB-468 cells, but not non-metastatic MCF-7 and T47D cells. Binding of exE-selectin/Fc was abolished by removal of tumor cell surface sialyl lewis x (sLe^x^) moieties. Employing an exE-selectin/Fc affinity column, we further purified the counterreceptor of E-selectin from metastatic breast cancer cells. The N-terminal protein sequence and cDNA sequence identified this E-selectin ligand as a ∼170 kD human CD44 variant 4 (CD44v4). Purified CD44v4 showed a high affinity for E-selectin via sLe^x^ moieties and, as expected, MDA-MB-231 cell adhesion to and migration across HUVEC monolayers were significantly reduced by down-regulation of tumor cell CD44v4 via CD44v4-specific siRNA.

**Conclusions/Significance:**

We demonstrated, for the first time, that breast cancer cell CD44v4 is a major E-selectin ligand in facilitating tumor cell migration across endothelial monolayers. This finding offers new insights into the molecular basis of E-selectin–dependent adhesive interactions that mediate breast cancer cell transendothelial metastasis.

## Introduction

Metastatic invasion is the primary cause of breast cancer mortality. A key step in the metastasis process is migration of tumor cells across the blood vessel-lining endothelial monolayers. It has been widely reported that endothelial cell E-selectin plays a pivotal role in mediating cell–cell interactions between tumor cells and endothelial monolayers during tumor metastasis [1], [2], [3]. The major ligand of endothelial E-selectin on the tumor cell surface has been identified as a sialylated glycan determinant, such as sialyl Lewis x moieties (sLe^x^), which decorate the terminal extensions of O-linked or N-linked carbohydrates [4]. Interaction of tumor cell surface sLe^x^ moieties and sLe^x^-decorated glycoproteins with endothelium E-selectin is a major component of cancer invasion and metastasis. A positive correlation between expression of E-selectin ligands such as sLe^x^ moieties in tumor cells and tumor cell metastasis or invasion has been widely reported [5], [6]. In breast cancer cells, several studies have also demonstrated a critical role for E-selectin in regulating tumor cell transendothelial migration [7], [8]. However, the identity of the E-selectin ligand in breast cancer cells and its physiological contribution in regulating tumor cell transendothelial migration is unknown.

Several leukocyte adhesion molecules, including leukocyte P-selectin glycoprotein ligand-1 (PSGL-1) and E-selectin ligand-1 (ESL-1), have been identified as ligands for endothelial E-selectin [9]. These interactions between PSGL-1, ESL-1 and E-selectin play a significant role in regulating leukocyte rolling process. Dimitroff et al. [10] reported that both E-selectin binding forms of PSGL-1 and ESL-1 are expressed on the human bone-metastatic prostate tumor MDA PCa 2b cell line, suggesting that these molecules may serve as E-selectin ligands in mediating tumor cell adhesion to or migration across endothelium. However, it is unclear whether breast cancer cells or other non-bone derived metastatic tumor cells express PSGL-1 or ESL-1. Recently, studies have demonstrated that CD44 variant isoforms (CD44v) in LS174T colon carcinoma cells possess selectin binding activity [11], [12], suggesting a broader role for CD44v in regulating tumor cell metastasis, particularly the event of migration across the vascular endothelium. CD44 was originally identified as a leukocyte homing receptor, and its globular amino-terminal domain contains hyaluronic acid (HA)–binding motifs and several potential glycosylation sites [13]. Through its interaction with hyaluronan, CD44 serves as an adhesion molecule in cell–substrate and cell–cell interactions, lymphocyte recruitment to inflammatory sites, and tumor metastasis [14], [15], [16], [17]. The size of the CD44 molecule ranges from the standard 85–95 kD form (CD44s) to larger variant isoforms of 200 kD or more due to RNA splicing and post-translational modifications [18]. Functional characterization of different isoforms of the CD44 family, however, is still limited. Many cancer cell types express high levels of specific variants of CD44 [19]. The animal model studies have shown that interfering with the binding of CD44 to its ligand inhibits local tumor growth and metastatic spread [20], [21]. During tumor metastasis, cells detach from the primary tumor, penetrate the basement membrane into the connective tissue, and invade adjacent organs structures, including blood vessels. Tumor cells are subsequently transported to metastatic sites through the blood stream. However, the mechanisms by which CD44 modulates the tumor cell transendothelial migration process are not fully understood.

In the present study, we demonstrated that metastatic breast cancer cells strongly express a ∼170 kD CD44 variant 4 (CD44v4). The expression level and localization pattern of CD44v4 in breast cancer cells is closely correlated with tumor cell migratory capability across TNF-α pre-activated HUVEC monolayers. Our results further showed that CD44v4 is highly decorated with E-selectin binding sLe^x^ moieties and serves as a major adhesive molecule in mediating breast cancer cell transendothelial metastasis.

## Results

### Breast cancer cell transendothelial migration is E-selectin dependent

**Figure 1:**
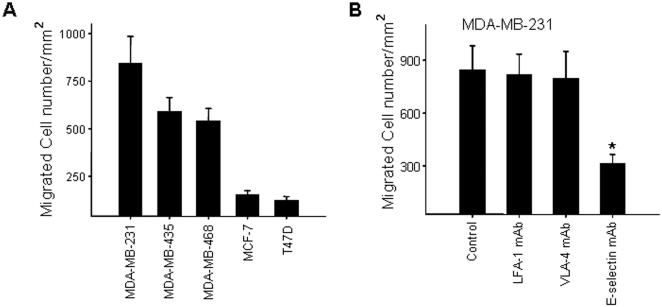
Transendothelial migration of breast cancer cells. A: differential migration of breast cancer cells across HUVEC monolayers pre-activated with TNF-α. B: migration of MDA-MB-231 across HUVEC monolayers in the presence of anti-E-selectin, anti-ICAM-1, and anti-VLA-4 antibodies (25 µg/ml each), respectively. HUVEC monolayers were pre-incubated with endothelial medium containing 25 ng/ml TNF-α for 6 h, and migration was terminated after overnight incubation at 37°C. All data are expressed as the mean±SD (*n* = 3) of a typical experiment and similar results were obtained in at least three separate experiments.

HUVEC monolayers cultured on collagen-coated permeable Transwell filters were used as a model for endothelial cell monolayers to assess the transmigration of breast cancer cells. HUVEC monolayers were pre-activated with 25 ng/ml TNF-α for 6 h to upregulate the expression of adhesion molecules before the tumor migration assay. Five breast cancer cell lines including MDA-MB-231, MDA-MB-435, MDA-MB-468, T47D, and MCF-7 were assessed for their migratory capability across HUVEC monolayers. The general migratory capability of these five breast cancer cells has been well-studied [15], [22]–[25]. Various breast cancer cells have a marked differential migratory capability. Among five cancer cell lines, MDA-MB-231, MDA-MB-435 and MDA-MB-468 have a higher invasive ability and are generally regarded as metastatic breast cancer cells while MCF-7 and T47D cells have a lower invasive ability and are regarded as non-metastatic cells. As shown in Figure 1A, MDA-MB-231 had the highest transmigration rate across TNF-α pre-activated HUVEC monolayers while MDA-MB-435 and MDA-MB-468 cells demonstrated relatively modest transmigration. Compared to these metastatic breast cancer cells, MCF-7 cells and T47D cells had a significantly lower transmigration rate. The transmigration of MDA-MB-231, MDA-MB-435 and MDA-MB-468 cross TNF-α pre-activated HUVEC monolayers was further characterized in the presence of various functional inhibitory antibodies. As shown in Fig. 1B, migration of MDA-MB-231 cells cross HUVEC monolayers was significantly blocked by anti-E-selectin antibody, but not by inhibitory antibodies against LFA-1 or VLA-4 (Fig. 1B), suggesting that metastasis of breast cancer cells across TNF-α pre-activated HUVEC monolayers is specifically dependent on HUVEC E-selectin. Similar inhibition by anti-E-selectin antibody on tumor cell transendothelial migration was observed in both MDA-MB-435 and MDA-MB-468 cell migration assays (data not shown).

**Figure 2:**
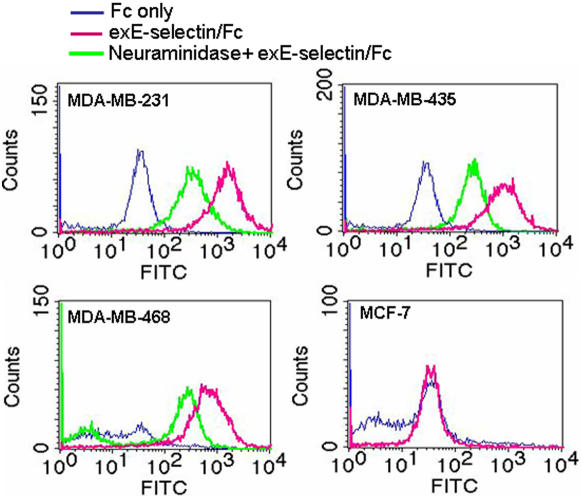
Binding of soluble exE-selectin/Fc recombinant to breast cancer cells via sLe^x^ moieties on the tumor cell surface. Trypsin-elicited breast cancer cells were surface labeled with soluble exE-selectin/Fc or Fc-only as detailed in the Methods. The bound Fc recombinants were detected by Alexa Fluor 488-conjugated goat anti-rabbit Fc and measured by FACS. In separated experiments, tumor cells were treated with neuraminidase to remove surface sLe^x^ moieties before exE-selectin/Fc binding.

Since the migration results suggest that E-selectin plays a critical role in mediating breast cancer cell transendothelial migration, we next examined the interactions of breast cancer cells with a soluble recombinant protein of E-selectin extracellular domain (exE-selectin/Fc). A direct binding assay of breast cancer cells with soluble exE-selectin/Fc was performed. In the experiment, exE-selectin/Fc or Fc-only (served as a control) was incubated with non-fixed breast cancer cells at 4°C for 1 h, and bound Fc was detected by fluorescently conjugated goat anti-rabbit Fc antibody. As shown in Figure 2, the exE-selectin/Fc recombinant strongly bound to MDA-MB-231, MDA-MB-435 and MDA-MB-468 cell surfaces. The Fc-only control did not bind to any breast cancer cells. In contrast to MDA-MB-231, MDA-MB-435 and MDA-MB-468 cells, MCF-7 cells showed no significant binding of exE-selectin/Fc recombinant. A similar negative staining of exE-selectin/Fc was observed in T47D cells (data not shown). Since sLe^x^ moieties are major binding partners of E-selectin, we further determined whether the binding of exE-selectin/Fc to metastatic breast cancer cells was dependant on tumor cell surface sLe^x^ moieties. As shown in the figure, removal of sLe^x^ moieties from the tumor cell surface by neuraminidase treatment markedly reduced the binding of exE-selectin/Fc. Taken together, these results suggested that sLe^x^ moieties are likely involved in mediating the binding interactions of breast cancer cells to E-selectin and that breast cancer cell transendothelial migration is positively correlated to the binding activity of the exE-selectin/Fc recombinant to tumor cells.

**Figure 3:**
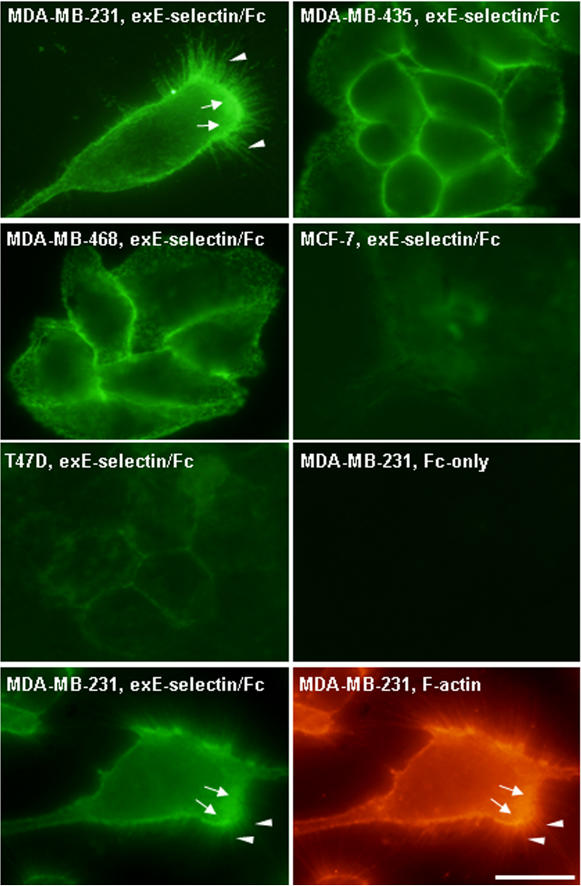
Localization of potential E-selectin ligand(s) on the breast cancer cell surface. A: After blocking with 5% normal goat serum in PBS, breast cancer cells cultured in a tissue culture dish were incubated with exE-selectin/Fc (10 µg/ml) in blocking solution for 1 h at 4°C. Following three washes, cells were fixed with 3.5% paraformaldehyde (5 min, 20°C) and the bound exE-selectin/Fc was detected by Alexa Fluor 488-conjugated anti-rabbit Fc. Cell surface labeling with Fc-only served as a background control. B: To determine the localization of E-selectin ligand on metastatic tumor cell surfaces during migration, MDA-MB-231 cells were double labeled with soluble exE-selectin/Fc for E-selectin ligand (left panel) and rhodamine-conjugated phalloidin for F-actin (right panel). Bar, 20 µm.

We next determined the localization pattern of E-selectin binding protein(s) on the breast cancer cell surface. Tumor cells cultured in tissue culture dishes were directly incubated with soluble exE-selectin/Fc or Fc-only for 1 h at 4°C. As shown in Figure 3, MDA-MB-231, MDA-MB-435 and MDA-MB-468 cells were all strongly labeled with exE-selectin/Fc, while MCF-7 and T47D were not or only weakly stained. This observation was in agreement with flow cytometry analysis (Fig. 2). However, among three metastatic breast cancer cells, the binding patterns of the exE-selectin/Fc recombinant were strikingly different. On the surface of highly metastatic MDA-MB-231 cells, exE-selectin/Fc bound strongly to the migrating front of the cell (arrows), particularly the “spiking” cellular protrusions (arrowheads). In contrast, exE-selectin/Fc was found to be concentrated at sites of cell-cell contact in both MDA-MB-435 and MDA-MB-468 cells, which had relatively less transmigration compared to MDA-MB-231 cells (Fig. 1). These results suggested that metastatic breast cancer cell transendothelial migration might also be correlated to the localization pattern of E-selectin binding protein(s) on the tumor cell surface. Co-staining MDA-MB-231 cells with exE-selectin/Fc recombinant and rhodamine-conjugated phalloidin showed that F-actin (red, arrows) was predominantly localized at the exE-selectin/Fc binding region (green, arrows), including the “spiking” cellular protrusions (arrowheads). The co-distribution of F-actin and the exE-selectin/Fc recombinant in metastatic breast cancer cells suggests that the E-selectin binding protein(s) were mainly localized to the leading edge or migrating front of MDA-MB-231 cells during metastasis.

### Identification of breast cancer cell CD44v4 as a major sLe^x^ moieties-decorated membrane glycoprotein that binds to E-selectin

**Figure 4:**
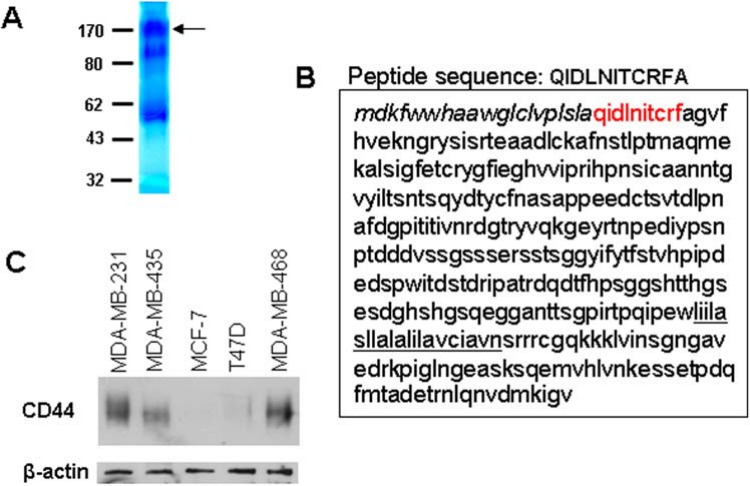
Identification of the major E-selectin binding protein in metastatic breast cancer cells as human CD44. A: Gel-code staining of proteins eluted from an exE-selectin/Fc affinity column. A 170 kD protein indicated by the arrow was selected as a potential ligand of E-selectin in tumor cells since 130 kD and 50 kD protein bands were confirmed as exE-selectin/Fc and IgG heavy chain, respectively. B: matching of the 10 amino acid N-terminal peptide sequence (red) of the 170 kD purified protein with that of human CD44. C: Western blot analysis of five breast cancer cell lines with anti-CD44H antibody.

In order to isolate and identify the E-selectin binding protein(s) that mediate breast cancer cell adhesion to and migration across endothelial monolayers, we constructed an affinity column by immobilizing exE-selectin/Fc to protein A-conjugated Sepharose. ∼5×10^8^ MDA-MB-231 cells were used to obtain sufficient quantities of E-selectin ligand for microsequence analysis. As shown in Figure 4A, three major bands around 170 kD, 130 kD and 50 kD were found in the eluted proteins. Among these three protein bands, the 50 kD and 130 kD bands were confirmed as IgG heavy chain and the exE-selectin/Fc recombinant, respectively. Thus, the protein band at 170 kD was selected for further analysis. The N-terminal peptide sequence of 10 amino acids, QIDLNITCRFA, from the 170 kD purified protein had a sequence that 100% matched the matured form of human CD44, a highly glycosylated membrane protein (Fig. 4B). The CD44 identity of this glycoprotein was further confirmed by Western blot analysis against anti-CD44H antibody, which can recognize all CD44 variants (Fig. 4C). Western blot results also confirmed that metastatic MDA-MB-231, MDA-MB-435 and MDA-MB-468 cells all strongly expressed CD44, while non-metastatic MCF-7 and T47D cells had little CD44 expression.

**Figure 5:**
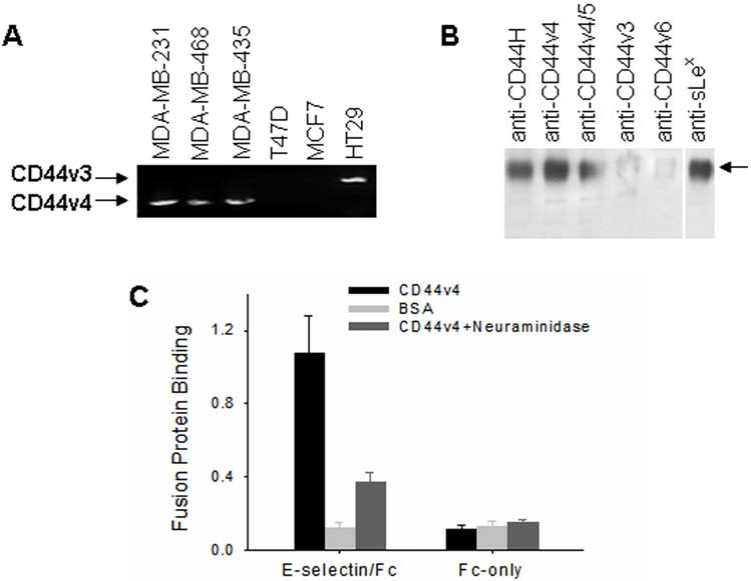
Identification of the CD44 variant isoform in metastatic breast cancer cells as CD44v4. A: RT-PCR amplification and DNA sequence of CD44 extracellular domain from various breast cancer cells and colonic HT29 cells. B: Western blot analysis of the 170 kD purified protein with antibodies specific to sLe^x^ moieties and different CD44 variant isoforms, respectively. C: Specific binding of the 170 kD purified protein (CD44v4) to E-selectin through sLe^x^ moieties. The data are represented as the mean±SD of three different experiments.

Since CD44 has multiple variants containing the common N- and C-terminal region but a different inserted portion within the extracellular domain due to alternative splicing, the N-terminal sequence alone can not distinguish which CD44 variant is involved in mediating breast cancer transendothelial metastasis. To solve this problem, we amplified the cDNA encoding the CD44 extracellular domain from various breast cancer cells using a pair of primers, forward primer 5′-TATAAGCTTTTCGCTCCGGACACCATGGACAAG-3′, and reverse primer 5′-ATAAGATCT TTCTGGAATTTGGGGTGTCCTTAT-3′. Since this pair of primers target the sequence shared by all CD44 variants, they amplify all variant isoforms of the CD44 protein. As shown in Figure 5A, a major band around 1.2 kb was observed in MDA-MB-231, MDA-MB-435 and MDA-MB-468. The DNA sequence of the 1.2 kb PCR product indicated that it was CD44 variant 4 (CD44v4) (gene accession number: NM001001391). No PCR product was amplified from either MCF-7 or T47D cells. Interestingly, a slightly larger PCR product was amplified from colonic HT29 cells using the same primers, and its sequence matched that of CD44 variant 3 (CD44v3) (gene accession number: NM001001390). To confirm CD44v4 identification by PCR, we blotted the 170 kD purified protein with a panel of monoclonal antibodies that specifically react with different variants of human CD44. As shown in Figure 5B, Western blot analysis confirmed the 170 kD protein as CD44v4 (Fig. 5B). Blotting the 170 kD protein band with anti-sLe^x^ moieties antibody also showed that this CD44 variant isoform was decorated with sLe^x^ moieties. Figure 5C shows the specific binding of purified CD44v4 to exE-selectin/Fc and that this binding occurred through sLe^x^ moieties on CD44v4.

### CD44v4 serves as a major E-selectin ligand in mediating breast cancer cell transendothelial metastasis

**Figure 6:**
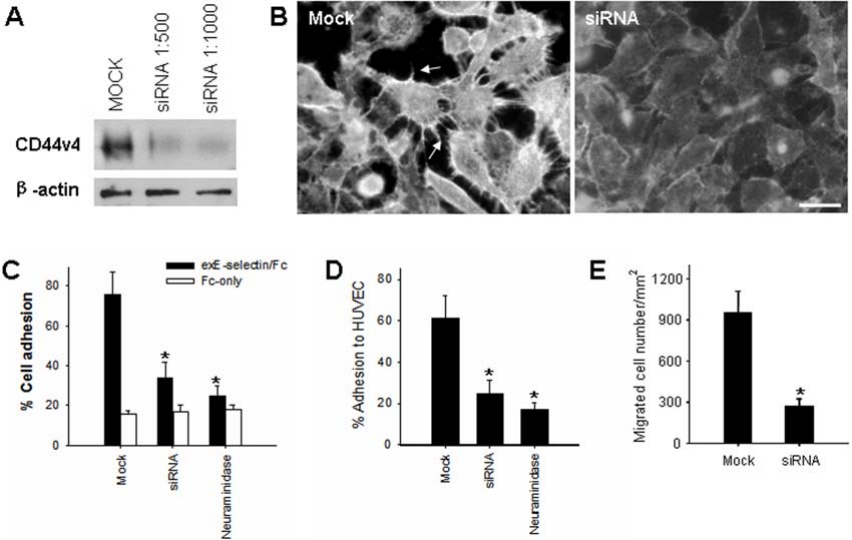
The role of CD44v4 in mediating breast cancer cell adhesion and transmigration. A and B: siRNA transfection-induced CD44v4 downregulation in MDA-MB-231 cells as shown by Western blot (A) and immunofluorescence labeling (B), respectively. C and D: reduction of MDA-MB-231 cell adhesion to immobilized recombinant exE-selectin/Fc (C) and TNF-α pre-activated HUVEC monolayers (D) after CD44v4 downregulation via siRNA. E: reduction of MDA-MB-231 cell transendothelial migration by CD44v4 downregulation. The data are representative of three independent experiments.

To characterize the role of CD44v4 in mediating breast cancer cell transendothelial migration, we next examined the effects of CD44v4 knockdown as well as removal of tumor cell surface sLe^x^ moieties on tumor cell adhesion to and migration across TNF-α pre-activated HUVEC monolayers. CD44v4 knockdown in breast cancer cells was achieved by transfecting MDA-MB-231 cells with CD44v4 siRNA [26]. As shown in Figure 6A, Western blot analysis demonstrated that the CD44v4 expression level was significantly decreased in MDA-MB-231 cells after CD44v4 siRNA transfection. However, CD44v4 protein level was not changed in MDA-MB-231 cells transfected with control oligonucleotides. A marked decrease in cell surface expression of CD44v4 after CD44v4 siRNA transfection was also shown by cell surface immunofluorescence labeling (Fig. 6B). Interestingly, CD44v4 siRNA transfection not only decreased the CD44v4 protein level on the MDA-MB-231 cell surface, but also changed tumor cell morphology. As can be seen in figure 6B, mock–transfected MDA-MB-231 cells maintained their typical spindle shape and had numerous “spiking” protrusions brightly labeled with anti-CD44H antibody (arrowheads). In contrast, in CD44v4 siRNA–transfected MDA-MB-231 cells, no anti-CD44H antibody labeled “spiking” cellular protrusions were observed, and tumor cells had lost their long spindle shape. The E-selectin binding and transmigration capacity of MDA-MB-231 cells before and after CD44v4 downregulation was further determined. As shown in Figure 6C, mock-transfected MDA-MB-231 cells strongly adhered to immobilized exE-selectin/Fc, with an adhesion rate of 79.5±10.2% of the total applied cells. However, the adhesion of CD44v4 siRNA-transfected MDA-MB-231 cells was significantly decreased, with an adhesion rate of 31.2±7.4%. The adhesion rate of CD44v4-downregulated MDA-MB-231 cells was close to that of MDA-MB-231 cells pretreated with neuraminidase (21.4±4.2%). Similar results were observed in adhesion assays using TNF-α pre-activated HUVEC monolayers instead of immobilized recombinant exE-selectin/Fc (Fig. 6D). CD44v4 siRNA–transfected MDA-MB-231 cells also showed a marked reduction in transmigration across TNF-α pre-activated HUVEC monolayers. As shown in Figure 6E, after overnight incubation, transendothelial migration of MDA-MB-231 cells transfected with CD44v4 siRNA was reduced to only one third of that of mock-transfected MDA-MB-231 cells.

## Discussion

Breast carcinoma is one of the most common tumors recently found in female patients, and its metastasis is a major cause of death. Although the metastasis of breast cancer cells is a complex process that requires the activity of multiple genes including urokinase plasminogen activator, cytokines, chemokines, and matrix metalloproteinases (MMPs) [27], [28], [29], it has been widely reported that the selectin family of adhesion molecules and their ligands play a critical role in tumor cell metastasis through blood vessels [1], [2], [3]. During tumor cell metastasis, binding interactions generally occurred between selectin-expressed endothelial cell monolayers and selectin ligands on tumor cells. The binding capacity of tumor cells to blood vessel-lining endothelium through E-selectin was closely correlated to the metastatic capability of breast cancer cells. A previous study demonstrated that E-selectin ligation regulated the transendothelial migration of colon cancer cells via mediating p38 and ERK MAP kinase activation [30]. However, E-selectin ligands expressed in migrating tumor cells, particularly breast cancer cells, are not well-characterized. In the present study, we show that the CD44 variant isoform 4 (CD44v4) is a major metastatic breast cancer cell glycoprotein decorated with sLe^x^ moieties and serves as an E-selectin ligand in facilitating tumor cell migration across TNF-α pre-activated HUVEC monolayers.

### CD44v4 mediates breast cancer cell adhesion to endothelial monolayers via interacting with E-selectin

In the present study, the binding activity of breast cancer cells to E-selectin was carefully characterized at the cellular level. Using soluble E-selectin/Fc recombinant as a probe, we directly detected the binding of E-selectin to living breast cancer cells. The results clearly demonstrated a positive correlation between the binding activity of soluble E-selectin/Fc recombinant and the ability of breast cancer cells to migrate across endothelial cell monolayers. Firstly, E-selectin binding protein(s) are abundantly expressed in metastatic breast tumor cells including MDA-MB-231, MDA-MB-435 and MDA-MB-468, but not in non-metastatic tumor cells such as MCF-7 and T47D (Fig. 2 and Fig. 3). Secondly, labeling of E-selectin/Fc recombinant on the breast cancer cell surface also demonstrated a strikingly different pattern among various metastatic tumor cells. As shown in Figure 3, in MDA-MB-231 cells, which had the highest transmigration rate across TNF-α pre-activated HUVECs, the E-selectin/Fc recombinant strongly bound to the migrating cell front, particularly the “spiking” structures of cell protrusions. In contrast, in MDA-MB-435 and MDA-MB-468 cells, which had relative lower transmigration rates compared to MDA-MB-231 cells, binding of the E-selectin/Fc recombinant was mainly found at sites of cell-cell contacts. Taken together, these results suggest that breast cancer cell transendothelial migration is closely correlated to both the expression level and localization pattern of cell surface E-selectin binding protein(s). In the present study, we selected HUVEC as a model endothelial cell line to characterize the interactions between breast cancer cells and endothelial monolayers during tumor metastasis is mainly due to the simplicity of HUVEC system. However, for better understanding breast tumor cell migration across blood vessel-lining endothelium under pathophysiologic conditions, primary cultured endothelial cells that are isolated from particular blood vessels should be used. Moreover, in order to mimic the real physiological situation of tumor metastasis, tumor cell transmigration across HUVEC monolayers pre-activated by TNF-α should be also measured under flow condition.

The binding of the E-selectin/Fc recombinant to metastatic breast cancer cells was largely reduced by removal of sLe^x^ moieties from tumor cell surfaces (Fig. 2 and Fig. 6C), suggesting that the E-selectin binding protein(s) in metastatic breast cancer cells are decorated with sLe^x^ moieties and that sLe^x^ moieties play a significant role in mediating tumor cell binding to E-selectin. Considering that sLe^x^ moieties are major E-selectin binding partner [31], this finding was not surprising. It is well-known that sLe^x^ moieties can mediate adhesion of tumor cells to the endothelium via interactions with E-selectin. In normal breast tissues, the expression levels of sLe^x^ moieties and sLe^x^ moiety-decorated glycoproteins are very low. However, up-regulation of sLe^x^ moiety expression has been widely reported to be correlated with poor prognosis and malignant relapse [5], [6].

By using an affinity column constructed by immobilizing E-selectin/Fc recombinant, the E-selectin binding protein that mediated breast cancer cell transendothelial migration was identified as human CD44 (Fig. 4). Further analysis based on RT-PCR and Western blot indicated it as CD44 variant 4 (CD44v4) (Fig. 5). As a multistructural and multifunctional cells surface adhesion molecule, CD44 is involved in cell-cell and cell-matrix interactions [13], [16]. There are multiple ligands of CD44, including the extracellular matrix (ECM) components collagen [32], fibronectin [33], laminin [34], chondroitin sulfate [35] and, particularly, hyaluronic acid (HA) [18]. Through binding all of these ligands, CD44 plays a critical role in initiating the metastatic spread of tumor cells [16], [36], [37]. Structurally, the genomic organization of CD44 is composed of twenty exons. Besides the first five and last five constant exons, there are 10 exons located between these regions that are subjected to alternative splicing, resulting in the generation of a variable region. Differential utilization of the 10 variable region exons, as well as variations in N- or O-glycosylation and glycosaminoglycanation (heparan sulfate or chondroitin sulfate), generates multiple CD44 isoforms. These isoforms have various tissue specificity and ligand binding activity. Some CD44 isoforms have been found to play an essential role in tumor progression [38]. Here, for the first time, our results demonstrate that CD44v4 is a major CD44 isoform expressed in metastatic breast cancer cells, and this CD44 variant is a membrane glycoprotein decorated with E-selectin binding sLe^x^ moieties.

### CD44v4 mediates breast cancer migration across endothelial cell monolayers

High levels of various CD44 variants (CD44v) are widely found in metastatic breast carcinoma [19], [38], [39]. This is confirmed by our observation that three metastatic breast carcinoma cell lines, MDA-MB-231, MDA-MB-435 and MDA-MB-468, expressed high levels of CD44v4 (Fig. 4 and 5). A number of lines of evidences have implicated CD44v with a possible role in tumor progression [40], [41]. However, how each CD44v contributes to the malignant phenotype, particularly the transmigration event of breast cancer cells across the endothelial monolayers lining the blood vessels, is largely unknown. Herrera-Gayol and Jothy [42] showed that CD44 variants (especially CD44v6) could regulate the binding of breast cancer cells to extracellular HA, and these binding interactions could mediate tumor cell metastasis. Recently, Napier et al [12] demonstrated that the LS174 colon carcinoma cell CD44 variant bound to selectin through sialidase-sensitive O-linked glycans presented on CD44. Here, we present evidence that CD44v4 is a major E-selectin ligand expressed in metastatic breast cancer cells, and plays a critical role in regulating tumor cell transendothelial invasion. In addition, binding of the E-selectin/Fc recombinant to the migrating front and particularly the “spiking” protrusions of MDA-MB-231 cells (Fig. 3) suggest that CD44v4 may be a key component of the tumor cell migration machinery. CD44v4 is likely involved in reorganization of the cell cytoskeleton during cell directional movement since CD44 variants can strongly associate with actin microdomains and ERM proteins [43]. CD44v4 may also regulate breast cancer cell transendothelial metastasis by functioning not only as an adhesive receptor for E-selectin, but also as a signaling transducer. It has been reported that phosphorylation of the CD44 isoform is required in tumor cell directional sensing of a phorbol ester gradient during invasion [44]. Engagement of CD44 has also been shown to induce protein kinase C and Rac activation during cell migration [45]. By pretreating breast cancer cells with a cross-linking reagent such as anti-CD44 antibody, Wang et al [15] reported that ligation of tumor cell surface CD44 enhanced tumor cell transendothelial migration by increasing the expression of lymphocyte function-associated antigen-1 (LFA-1) and very late antigen 4 (VLA-4). Similar up-regulation of tumor cell integrins and integrin-mediated adhesion by CD44 ligation was also found in a colon cancer cell line [46]. One possible mechanism by which CD44 ligation enhances integrin expression and tumor cell adhesion is through up-regulation of c-Met [46]. In the present study, however, we did not find a significant inhibitory role for either anti-LFA-1 antibody or anti-VLA-4 antibody during CD44v4-mediated breast cancer cell migration across TNF-α pre-activated HUVEC monolayers (Fig. 1), strongly arguing for a minor role of leukocyte-specific integrins in breast cancer cell metastasis.

In summary, the present study has demonstrated that CD44v4 is a major E-selectin ligand expressed in metastatic breast cancer cells and plays an essential role in regulating tumor cell adhesion to and migration across endothelial monolayers. The results expand our present knowledge of E-selectin-based tumor cell transendothelial metastasis, which might shed some light on the search for new pharmacological approaches to control breast tumor metastasis.

## Materials and Methods

### Cells

The human breast carcinoma cell lines, MDA-MB-231, MDA-MB-435S, MDA-MB-468, T47D, and MCF-7 were obtained from Dr. Paul Wade (Department of Pathology and Laboratory Medicine, Emory University, Atlanta) and Dr. Xiao Han (Nanjing Medical University, Nanjing, China). Breast cancer cells were grown in Dulbecco's modified Eagle's medium (Gibco BRL, Grand Island, NY) supplemented with 10% fetal bovine serum (Hyclone, Logan, UT), 100 units/ml penicillin and 100 µg/ml streptomycin. Human umbilical vein endothelial cells (HUVECs) were purchased from the China Cell Culture Center (Shanghai, China) and cultured in endothelial medium (Clontech, Mountain View, CA) supplemented with penicillin-streptomycin (100 U/ml and 100 µg/ml, respectively). All media were referred to as complete medium and all of the cell lines used in the study were free of *Mycoplasma*.

### Antibodies and Reagents

The monoclonal antibodies used in the experiments and their resources: mouse anti-human E-selectin mAb (MAB575), mouse anti-human CD44H (clone 2C5), mouse anti-human CD44v3 (clone 3G5), mouse anti-human CD44v6 (clone 2F10) and mouse anti-human CD44v4/5 (clone 3D2) (R&D Systems, Minneapolis, MN); Anti-CD44v4 mAb (Lab Vision, Fremont, CA); Anti-human LFA-1 and anti-human VLA-4 mAbs (BD Pharmingen, San Diego, CA); Anti-sLe^x^ mAb (Clone KM93) (Chemicon, Temecula, CA); HRP-conjugated goat anti-mouse and anti-rabbit IgG (Jackson ImmunoResearch Lab, West Grove, PA); Alexa Fluor 488(495/519) and Alexa Fluor 568(578/603)-conjugated secondary antibodies (Molecular Probes, Eugene, OR). BCECF-AM, rhodamine-conjugated phalloidin and ProLong antifade reagent were also obtained from Molecular Probes. TNF-α was purchased from Genetech (South San Francisco, CA).

### Soluble Human E-selectin-Fc Chimera Preparation

cDNA encoding the human E-selectin extracellular domain (residues 1-556) [47] was amplified from a human colon cDNA library (Clontech) by PCR using forward primer 5′-ATAT CATATGAAAAGAACTCTTGAAGTCATG-3′ and reverse primer 5′-ATATAGATCTAGCTTCACAGGTAGGTAGCAG-3′. The cDNA encoding the E-selectin extracellular domain was fused to a modified rabbit IgG1 Fc region and cloned into pcDNA3.1 (Invitrogen, Carlsbad, CA) using the *NdeI*/*HpaI* digestion site. COS-7 cells were used for transfection and fusion protein generation [48]. E-selectin/Fc was affinity purified from cell culture supernatants by Protein A-Sepharose (Sigma, St. Louis, MO) and eluted with 100 mM glycine/HCl, pH 3.5, immediately followed by neutralization, concentration and dialysis. As a control, the Fc portion of the fusion protein was also prepared [48]. Commercial Recombinant Human E-selectin/CD62E/Fc chimera was obtained from R&D Systems.

### Immunofluorescence and Flow Cytometric Analysis

For breast cancer cell surface labeling with soluble exE-selectin/Fc, cells grown to confluence on a culture dish were washed three times with cold HBSS and blocked with 5% normal goat serum in HBSS for 30 min on ice. Soluble exE-selectin/Fc and Fc-only (10 µg/ml each) were dissolved in blocking buffer and added to the cell culture dishes. After 1 h incubation on ice, cells were washed three times with HBSS and fixed with 3.7% paraformaldehyde (5 min, 20°C). The bound exE-selectin/Fc or Fc-only was detected by fluorescence conjugated goat anti-rabbit Fc antibody. In some experiments, cells labeled with exE-selectin/Fc were further permeabilized with 0.03% Triton X-100 (5 min, 4°C) and stained with rhodamine conjugated phalloidin (30 min, 4°C). After extensive washing, labeled cells were then mounted in ProLong antifading embedding solution and analyzed using a fluorescence microscope equipped with an imaging system (Olympus, Japan). A 100×NA/1.3 Plan-Neofluar oil immersion lens (Olympus) was used for all images. Images shown are representative of at least three experiments, with multiple images taken per slide. As a control for background labeling, cells were incubated with comparable concentrations of irrelevant IgG and secondary antibody. For flow cytometric analysis, suspended tumor cells (1×10^7^ cells; 1 ml) incubated with E-selectin/Fc fusion protein or Fc only were fixed and then incubated for 45 min at 4°C with Alexa Fluor 488-conjugated antibody solution (1:1000 dilution). After three washes, cells were re-suspended and flow cytometry analysis was performed on a FACS instrument. The results were analyzed using CELLQUEST software (BD Biosciences).

### E-selectin Ligand Purification and Identification

An affinity column was constructed by immobilizing exE-selectin/Fc to protein A-conjugated Sepharose beads. ∼500 cm^2^ of confluent MDA-MB-231 cells were used for E-selectin ligand isolation. Proteins were eluted from the affinity column at low pH (150 mM NaCl, 100 mM glycine/HCl, pH 3.5, containing 1% *n*-octylglucoside). The eluant was pH-neutralized and resolved by 10% SDS-PAGE, and the protein bands were visualized by Gelcode 250 staining. The resulting ∼170 kD protein band (∼100 pmol) was extracted and subjected to direct N-terminal protein sequence analysis.

### Western Blotting

Cell lysates were homogenized in loading sample buffer, normalized for total protein, and loaded on 10% SDS-PAGE gels. After electrophoresis and transfer onto Hybond nitrocellulose membranes (Amersham, Piscataway, NJ), membranes were blocked with 5% non-fat milk in TTBS, incubated with primary antibody followed by incubation with horseradish peroxidase–conjugated secondary antibody and ECL detection (Amersham). The sLe^x^ moieties were detected by mouse anti-sLe^x^ antibody, while CD44v was detected using a panel of monoclonal antibodies against various CD44 isoforms including CD44H, respectively.

### siRNA Experiment

The CD44v4 siRNA experiment was performed as previously described [26]. CD44v4 siRNA oligonucleotides, antisense 5′-UAUAUUCAAAUCGAUCUGCTT-3′ and sense 5′-GCAGAUCGAUUUGAAUAUATT-3′ (GC Content: 31.6%, position 494), were obtained from Sigma. Briefly, annealed siRNA oligonucleotides were transfected into MDA-MB-231 cells seeded in tissue culture plates (35–50% confluent) with 100 µM Lipofectamine 2000 (Invitrogen) in Opti-MEM reduced-serum medium (Invitrogen) [26]. Cells were incubated at 37°C for 4 hours before addition of complete medium and cultured for an additional 72 hours before further assays. In the mock transfection control group, oligonucleotides (5′-UCUACUCUUCCUUCUGCAACCCTT-3′ and 5′-GGGUAGCAGAA GGAGUAGATT-3′) (Sigma) were used.

### Protein Binding Assay

Purified CD44v4 protein (∼150 µg/ml in the final solution containing 1% *n*-octylglucoside) was diluted 1∶20 with PBS and 50 µl of diluted solution was immediately added to each well of a 96-well microtiter plate (Costar, Cambridge, MA). Plates were incubated overnight at 4°C to allow protein immobilization. Wells coated with 1% BSA served as controls. After blocking with 1% BSA, plates were incubated with exE-selectin/Fc or Fc only (5 µg/ml each) in blocking solution for 1 h at 37°C. After three washes, bound Fc recombinant was detected by HRP-conjugated goat anti-rabbit Fc antibody and OD measurement. In some experiments, immobilized CD44v4 was treated with neuraminidase for 30 min at 37°C to remove sLe^x^ moieties prior to the Fc-recombinant binding assay.

### Cell Adhesion Assay

The adhesion assay was performed in 96-well plates as previously described [46], [49], [50]. For cells adhesion to immobilized recombinant E-selectin, 100 µl exE-selectin/Fc or Fc-only (20 µg/ml each) was added to a 96-well microtiter plate and incubated overnight at 4°C to allow protein immobilization. Plates were then blocked with 1% BSA in HBSS for 30 min. For cell adhesion to HUVEC monolayers, 2×10^5^ HUVECs cells were grown to confluence on collagen-coated Transwell membranes, and then treated for 6 h with 25ng/ml TNF-α [51], [52]. Suspended breast cancer cells were pre-labeled with 2.5 µM BCECF-AM for 10 min at 37°C. After three washes, cells were re-suspended in HBSS containing 1% BSA at a concentration of 2×10^5^ cells/ml. 150 µl of the cell suspension was added to 96-well plates or the upper chamber of Transwell setups. After 1 h incubation in the presence of various antibodies at 25 µg/ml each, plates and Transwell filters were washed three times to remove unbound tumor cells. The fluorescence of plates was read directly on a Fluorescence Measurement System (Millipore, Cytofluor 2300) at wavelengths of 485/538 nm excitation/emission. For Transwell setups, cells were elicited by trypsin/EDTA and re-suspended in 150 µl HBSS for fluorescence measurement.

### Transmigration assay

Transendothelial migration assays of breast cancer cells were performed as described previously with a minor modification [53]. Briefly, 5.0 µm pore-size Transwell filters were coated with rat collagen at a concentration of 150 µg/cm^2^ by drying overnight under a laminar flow hood. HUVECs were seeded onto rehydrated coated membranes at a concentration of 2×10^5^ cells/well. After confluent monolayer formation, HUVEC monolayers were treated with 25 ng/ml TNF-α for 6 h [51], [52]. Invasion assays of breast cancer cells were performed by applying approximately 10^6^ disaggregated cells to the upper reservoir. The invasion medium was placed in the wells on both sides of the membranes. Invasion assays were incubated overnight at 37°C and, thereafter, HUVEC monolayers and non-invading cells on the upper surface of the membrane were removed. Migrated tumor cells on the bottom side of the membranes and in the lower chamber were collected, stained and calculated under a microscope [15].

### Statistics

The data are expressed as the mean±SD of at least three experiments performed in quadruplicate. Statistical analysis of the significance of differences between groups was carried out using a Student's *t* test. A *p*<0.05 was considered significant.
